# Exosome secretion and cellular response of DU145 and PC3 after exposure to alpha radiation

**DOI:** 10.1007/s00411-022-00991-5

**Published:** 2022-09-13

**Authors:** Beata Pszczółkowska, Wioletta Olejarz, Mateusz Filipek, Adrianna Tartas, Grażyna Kubiak-Tomaszewska, Aleksandra Żołnierzak, Katarzyna Życieńska, Józef Ginter, Tomasz Lorenc, Beata Brzozowska

**Affiliations:** 1 Biomedical Physics Division, Faculty of Physics, University of Warsaw, 5 Pasteura Street, Warsaw, 02-093 Poland; 2 Department of Biochemistry and Pharmacogenomics, Medical University of Warsaw, 1 Banacha Street, Warsaw, 02-097 Poland; 3 Laboratory of Centre for Preclinical Research, Medical University of Warsaw, 1 Banacha Street, Warsaw, 02-097 Poland; 4 1st Department of Clinical Radiology, Medical University of Warsaw, 5 Chałubińskiego Street, Warsaw, 02-004 Poland

**Keywords:** Exosomes, High LET radiation, Alpha particles, Prostate cancer

## Abstract

Exosomes are spherical membrane nanovesicles secreted from cells, and they play an important role in tumor immune response, metastasis, angiogenesis, and survival. Studies investigating exosomes isolated from cells exposed to photon radiation commonly used in conventional radiotherapy demonstrate the influence of this type of radiation on exosome characteristics and secretion. There is currently no research investigating the effects of densely ionizing particles such as protons and alpha radiation on exosomes. Thus we have evaluated the cellular response of human prostate cancer cells exposed to 0, 2, and 6 Gy of alpha radiation emitted from the Am-241 source. Irradiated PC3 and DU145 cell lines, characterized by differences in radiosensitivity, were studied using apoptosis, LDH, and IL-6 assays. Additionally, the corresponding concentration and size of isolated exosomes were measured using NTA. We found that exposure to ionizing radiation resulted in gross changes in viability and cell damage. There were increased amounts of apoptotic or necrotic cells as a function of radiation dose. We demonstrated that irradiated PC3 cells secrete higher quantities of exosomes compared to DU145 cells. Additionally, we also found no statistical difference in exosome size for control and irradiated cells.

**Supplementary Information:**

The online version contains supplementary material available at 10.1007/s00411-022-00991-5.

## Introduction

Prostate cancer is one of the most common cancers among men in Western countries (Culp et al. 2020). Diagnostic techniques include a tissue biopsy and/or a prostate-specific antigen (PSA) blood test. However, these two detection methods are not sensitive enough to diagnose the early stages of prostate cancer and some aggressive tumors (Hoffman 2011). Current studies focus on developing new biomarkers for the diagnosis and prognosis of prostate cancer. Researchers are now considering minimally invasive liquid biopsies to glean new information about prostate cancer. Liquid biopsies refer to the analysis of urine, blood, and other human body fluids to characterize important information about a specific tumor such as circulating tumor cells (CTCs), cell-free tumor DNAs (ctDNAs), and extracellular vesicles (EVs) (Merker et al. 2018; Gao et al. 2021). Cells secrete three main types of EVs: exosomes, microvesicles, and apoptotic bodies based on their differences in biogenesis and size (György et al. 2011).

Exosomes are spherical, double-membrane nanovesicles ranging from 30 to 150 nm in diameter and density varying between 1.1 and 1.9 g/cm$^{3}$ (Zhang et al. 2018). They contain various cargoes such as miRNAs, mRNAs, proteins, lipids, and viral particles (Pan et al. 2017). They are released mostly by eukaryotic cells, both healthy and unhealthy, and are involved in cell-to-cell communication (Dominiak et al. 2020), angiogenesis (Olejarz et al. 2020a) and immunosuppression (Olejarz et al. 2020b). EVs are divided into three main types: exosomes, microvesicles, and apoptotic bodies based on differences in diameter, size, and biogenesis. Exosomes originate by the inward growth of the plasma membrane to produce early endosomes. Membranes of the early endosome partially invaginate and bud into the lumen with cytoplasmic content to form intraluminal vesicles (ILVs, precursors of exosomes). Late endosomal structures that accumulate dozens of ILVs mature and are known as multivesicular bodies (MVBs). MVBs migrate to the cell surface and then fuse to the plasma membrane and release their vesicular content (exosomes) into the extracellular space. Therefore, exosomes are the only EV generated by being released into the extracellular space by exocytosis (Vlaeminck-Guillem 2018; Yue et al. 2020). Exosomes are involved in tumor progression and promoting tumor cell migration during metastasis (Lorenc et al. 2020). EVs can be transformed by mutant p53 proteins into oncogenic messengers that reprogram tumor-host communication within the entire organism to promote the dissemination of metastatic tumor cells. Tumor progression to a final lethal and metatstatic stage is dependent on a tumor-supporting microenvironment that is induced by communication between tumor and host stromal cells. The crucial mediators of tumor-stroma communication are exosomes, which operate both locally within the primary tumor and in distant organs (Pavlakis et al. 2020). Tumor-stroma crosstalk is caused by the genetic changes in tumor cells. It turns out that the most common mutations are those in the TP53 gene that are correlated with metastasis, drug resistance, and poor patient survival. The p53 transcription factor is a critical element in the cell’s ability to regulate the cell cycle. Tumor protein p53 (product of the TP53 gene) expression increases in response to cellular stresses, such as ionizing radiation (IR), that cause DNA damage. Thus, the functional status of the p53 tumor suppressor is important in the progression of prostate cancer and dictates the overall effectiveness a given drug would have in disease treatment (Chappell et al. 2012). p53 functionality can also have a significant influence in determining the effect of alpha radiation on treatment efficiency.

Exosomes are described as critical mediators of several biological processes associated with tumor initiation and progression (Bastos et al. 2018). Researchers have demonstrated that exosomes play an important role in tumor immune response, metastasis, angiogenesis, and survival of migratory transformed cells (mesenchymal cells) into the circulation. These exosomes as well as those secreted by hypoxic cells transmit signals to endothelial cells to stimulate angiogenesis (Jelonek et al. 2016; Gulei et al. 2018). Liquid biopsies detect specific biomarkers in human body fluids. These biomarkers may be exosomes. A liquid biopsy of blood to collect exosomes can reveal the molecular characteristics of tumors, thus paving the way for the development of new therapies or optimizing radiotherapy methods.

Radiation therapy is a principal cancer treatment, with 50–60$\%$ of patients treated using this option (Delaney et al. 2005). The delivered radiation dose to the tumor is restricted to avoid toxicity to surrounding normal tissues. Moreover, restricting the delivered radiation dose often leads to unwanted effects such as suboptimal tumor control or deterioration of the quality of life (Barnett et al. 2009). As a result, a more in depth understanding of the tumor response to IR is necessary to determine the efficiency of radiation therapy and develop strategies for cancer cell radiosensitization. IR damages all cellular macromolecules and affects intercellular communication through different signal transduction systems. EVs are identified as significant mediators in intercellular communication. During cancer development, the signal transmission between cells plays an important role in tumor formation, metastasis, and progression (Archer et al. 2020).

The biological effectiveness of radiation related to DNA damage is determined by the dose delivered to the cells. The uniformity of the dose distribution depends on the ionization pattern. A uniform dose of cell irradiation means that the average amount of energy deposited inside a cell nucleus is the same for any kind of IR. However, the dose can be delivered with different spatial distribution (Friedland et al. 2011). The ionization density is represented by linear energy transfer (LET, given in keV per $\mu$m) and differs for low and high LET radiation. Gamma radiation and X-rays exhibit low LET and sparsely interact with the cellular environment inducing simple DNA damage. Conversely, alpha particles and heavy ions are characterized by high LET values which densely ionize the cell nucleus leading to complex DNA damage (Cheng et al. 2018). Radiation therapy with alpha-emitting radioligands has already demonstrated promising results in prostate cancer treatment (Kratochwil et al. 2016). Targeted alpha therapy selectively delivers radiation to prostate cancer cells while minimizing systemic toxic effects and represents an emerging treatment approach for prostate tumors.

The influence of indirect IRs such as $\gamma$-rays and X-rays on cancer cells and the exosomes secreted from them have been well studied (Yu et al. 2006; Lehmann et al. 2008; Arscott et al. 2013; Jella et al. 2014; Al-Mayah et al. 2015; Jelonek et al. 2015). However, the number of studies investigating the influence of charged particle beams, which ionize directly and have a high ionization density, on cancer cells and the exosomes secreted from them, is limited and does not include human prostate cancer cells. Therefore, we determined whether alpha particles induce changes in the profile of exosomes released from PC3 and DU145 cells after IR exposure to doses between 2 and 6 Gy. We investigated whether alpha radiation has functional consequences on exosome secretion and cellular response of human prostate cancer cells line of different radiosensitivity. The aim of this study was to assess the cytotoxic activities of alpha irradiation on the cellular environment, including the secreted exosomes. Alpha particles are characterized by high LET resulting in increased relative biological effectiveness (RBE). RBE is an empirical value that varies depending on several factors including the type of IR, the total dose, the dose rate, and the biological effects being considered such as cell death. Apoptosis is a programmed form of cell death that involves degradation of the cellular components such as DNA, the Golgi, the endoplasmic reticulum, and caspases. Following exposure to IR, reactive oxygen species (ROS) and free radicals are generated that induce DNA lesions which might promote apoptosis via activation of the caspase cascade (Chipuk et al. 2006). Irradiated cells can upregulate death receptors and make them sensitive to death, which can be investigated by studying the irradiated cell membrane and inflammatory system. The release of lactate dehydrogenase (LDH) can be regarded as a key marker of the prognosis of cancer patients, as a predictor of the cellular response (including cell radiosensitivity) to radiotherapy (RT) (Koukourakis and Giatromanolaki 2019). Mammalian cells exhibit different levels of radiosensitivity. While for some cells 2 Gy can lead to cell death or cell membrane damage, other more radioresistant cells require a higher radiation dose to achieve the same result. Therefore the efficiency of RT may be affected when the neoplastic lesion consists of cells with different radiation sensitivities or when cells become resistant to therapeutic radiation. In the tumor microenvironment, interleukin-6 (IL-6) plays an important role in cell apoptosis and the dissemination of solid tumors. IL-6 has a pathogenic role in the progression of prostate cancer and therefore represents a key target for cancer therapies (Santer et al. 2010). Exosome biogenesis is enhanced in cancer. Notably, tumor cells produce and secrete many more exosomes compared to normal proliferating cells (Szczepanski et al. 2011). Because exosomes are responsible for intracellular communication, researchers can investigate their role in the radiosensitization of human prostate cancer cell lines following IR.

## Materials and methods

### Cell culture

Human prostate cancer cell lines PC3 and DU145 were acquired from the American Type Culture Collection (ATCC CRL-1435 and HTB-81, respectively). PC3 cells were cultured in Dulbecco’s modified Eagle medium (DMEM-F12, biowest, Cat #L0092-500) supplemented with 10$\%$ fetal bovine serum (FBS, biowest, Cat #S1810-500) and 2.0$\%$ penicillin-streptomycin (AppliChem GmbH, Cat #A8943). DU145 cells were cultured in RPMI-1640 medium (biowest, Cat #L0498-500) supplemented with 10$\%$ FBS and 2.0$\%$ penicillin-streptomycin. Cells were grown in T-75 flasks as a monolayer and were incubated in a humidified atmosphere at 37$^\circ$C with 5$\%$ CO$_{2}$. Subculture was routinely performed when cells were 75–80$\%$ confluent using 0.25$\%$ trypsin-EDTA (biowest, Cat #X0930-100) at a ratio of 1:5.

### Irradiation

Two days prior to irradiation, cells were seeded onto glass coverslips which were then placed in a 6-well plate. Cells were seeded at a density of 2.0 $\times$ 10$^{5}$ cells/well in  5 mL of culture medium (DMEM-F12 or RPMI-1640 supplemented with 10$\%$ FBS and 2$\%$ penicillin-streptomycin). Cells were counted using automated cell counter EVE$^{TM}$ (NanoEnTek Inc.). Two replicates for each dose (including control cells) were investigated within a single experiment. Each experiment was performed independently three times in total for each cell line. In a separate experiment, 2.0 $\times$ 10$^{5}$ cells were seeded on two glass coverslips and then collected to obtain an exosome concentration sufficient for further analysis.

**Fig. 1:**
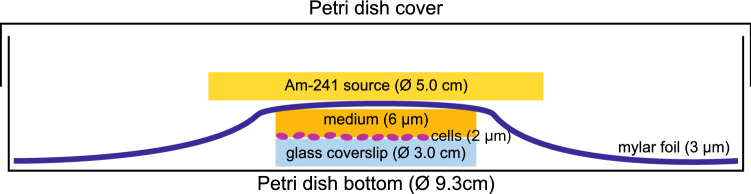
Cell irradiation setup with Am-241 flat source (not to scale)

Seeded cells were irradiated using the alpha radiation emitted from a flat source of Am-241 (AM1AP1 0053U, Eckert & Ziegler, Belgium). The source disc consists of two layers. A 0.1 µm thick gold layer covers the active layer (americium, 0.4 µm thick) with an activity of 100 kBq/cm$^{3}$, as specified by the producer. The dosimetric characteristics of this source is described in more detail elsewhere (Szefliński et al. 2020). The dose rate delivered to the cells and LET of alpha radiation was calculated based on Geant4 simulations. The mean LET of alpha radiation was equal to 135 ± 35 keV/µm (median 123 keV/µm). The dose rate was 0.6 Gy/min taking into account the irradiation geometry, which is shown in (Fig. 1).

Each glass coverslip was 30 mm in diameter and was placed inside a Petri dish that was 93 mm in diameter. Complete culture medium was added to the cells seeded on the coverslip (4 µL of medium corresponds to approximately 6 µm thickness over the coverslip). After covering the cells (2 µm thick monolayer) with mylar foil (3 µm thick), the source of alpha particles was put directly on top. The Petri dish was closed and placed in the incubator for a given amount of time according to the estimated dose rate. The prostate cancer cells were exposed to 2 and 6 Gy of irradiation. The irradiation time was equal to 3 min and 20 s and 10 min for 2 and 6 Gy, respectively.

After irradiation, the glass coverslip was put into a Petri dish with fresh medium (18 mL) without FBS (only DMEM-F12 or RPMI-1640 + 2.0$\%$ penicillin-streptomycin) and placed immediately in the incubator. After 96 h of incubation, the irradiated cells were analyzed and the supernatant solutions were collected and used for exosome isolation. The control and irradiated cells were cultivated in the serum-free medium for 4 days and then used for apoptosis, LDH, and IL-6 studies or exosome isolation. No significant differences were observed in cell proliferation when the medium was changed. It was important to use serum-free medium for exosome isolation to prevent measuring the exosomes that were secreted before irradiation and those that were present in the FBS.

### Exosome isolation

Exosomes were isolated from a medium conditioned by PC3 and DU145 cells using the differential centrifugation protocol proposed by Ludwig et al. (Ludwig et al. 2019a). In the first stage, culture supernatants were centrifuged at 2000 $\times$ g for 10 min at room temperature (RT) and at 10,000 $\times$ g for 30 min at 4$^o$C. Next, the culture supernatants were passed through a sterile syringe filter with a pore size of 0.22 µm (Millex, MilliporeSigma, SLGP033RS). Preconditioned supernatants were concentrated from 16–17 mL to 1 mL on Vivacell 100 concentrators (100,000 MWCO, Sartorius, VS2041). An aliquot (1 mL) of concentrated supernatants was added to a mini-SEC column (Econo-Pac chromatography columns, Bio-Rad, 7321010) to elute #0 fractions. After that, 1 mL of Dulbecco’s Phosphate Buffered Saline (PBS, Gibco, Cat #14190-136) was added to columns to wash out fraction #1. The same steps were repeated up to fraction #4 and this fraction was collected in an Eppendorf tube and used for exosome analysis.

### Apoptosis assay

An apoptosis detection kit (FITC:Annexin V Apoptosis Detection Kit I; BD Biosciences Pharmingen) was used to assess cell apoptosis. The apoptosis assay was performed according to the manufacturer’s protocol. The cells were analyzed (10,000 cells per sample) with a FACSCalibur flow cytometer (Becton Dickinson) using CellQuest Software.

### LDH assay - cell damage assessment

LDH released into cell culture media by dead cells was measured using the LDH Cytotoxicity Detection Kit (Takara Bio Inc.) according to the manufacturer’s instructions. The cell culture supernatants were pipetted into 96-well plates and recommended reagents (such as the Catalyst and the INT) were added. After 30 min of incubation at RT, the absorbance was measured at 490 nm using a microplate reader (Epoch microplate reader, BioTek Inc., USA) equipped with Gen5 software (BioTech Instruments, Inc., Biokom). Two measurements were taken for each replicate and the mean value was calculated. The maximal LDH release (${A_{high~control~sample}}$) was measured for non-irradiated cells lysed with 1$\%$ Triton X-100 prior and the obtained value was equal to 2. To estimate the minimal LDH release, the measurements were performed using non-irradiated cells (${A_{low~control~sample}}$) which were not treated with 1$\%$ Triton X-100 prior. Cytotoxicity (*C*) was calculated based on the absorbance measurements performed for irradiated cells (${A_{test~sample}}$) using the following equation:

1 $$\begin{aligned} C = \frac{A_{test~sample} - A_{low~control~sample}}{A_{high~control~sample} - A_{low~control~sample}} \cdot 100\% \end{aligned}$$

### Interleukin-6

IL-6 concentration was measured using an IL-6 ELISA kit purchased from Diaclone SAS (Besancon Cedex, France). To assess IL-6 level in cell culture the supernatant was analyzed using an enzyme-linked immunosorbent assay in accordance with the manufacturer’s protocol.

### Exosome studies with NTA

Nanoparticle tracking analysis (NTA) was performed by ZetaView (Particle Metrix, Germany) and its corresponding software (version 2.3) was used to assess the sizes (hydrodynamic diameters) and quantities of exosomes released from irradiated and non-irradiated prostate cancer cell lines. Before sample measurements, the instrument was calibrated using 100 nm polystyrene nano standard particles. Pre-acquisition parameters were set to a sensitivity of 85, a frame rate of 30 frames per second (fps), and a shutter speed of 100. The resulting average counted particles per frame was equal to 275, which was in agreement with previous work (Bachurski et al. 2019), where the sufficient particle per frame value was estimated to be 140–200 particles/frame. The dilution buffer (PBS) used for exosome samples was checked to ensure that the background number of vesicles in the NTA chamber was neglected. Before each measurement, a 1 mL syringe of PBS was injected into the chamber and no signal was detected (no light scattering) meaning it was free from particles. PBS was also used to clean the chamber between samples with different dilutions as was done previously (Gardiner et al. 2013). For each replicate, 2 mL of the isolated exosome sample (fraction #4) (Ludwig et al. 2019b), diluted in PBS, was loaded into the NTA chamber, and the measurements were taken at 11 different positions across the chamber, with two cycles of readings at each position. After automated analysis of all 11 measurements and removal of outliers, the mean, median, and mode (indicated as diameter) sizes, as well as the concentration of the sample, were calculated. Additionally, the exosome confirmation was performed using the NTA measurement with anti-CD9, -CD81, and -CD63 antibodies (Gardiner et al. 2013; Ludwig et al. 2019a). These results are included in supplementary materials.

### Statistical analysis

The results are represented as the mean values and their standard deviations calculated from three independent experiments performed in duplicate (each experimental measurement included two replicates). Comparisons were made using Student’s t-test assuming a significant level of 5$\%$.

## Results

### Apoptosis

**Fig. 2:**
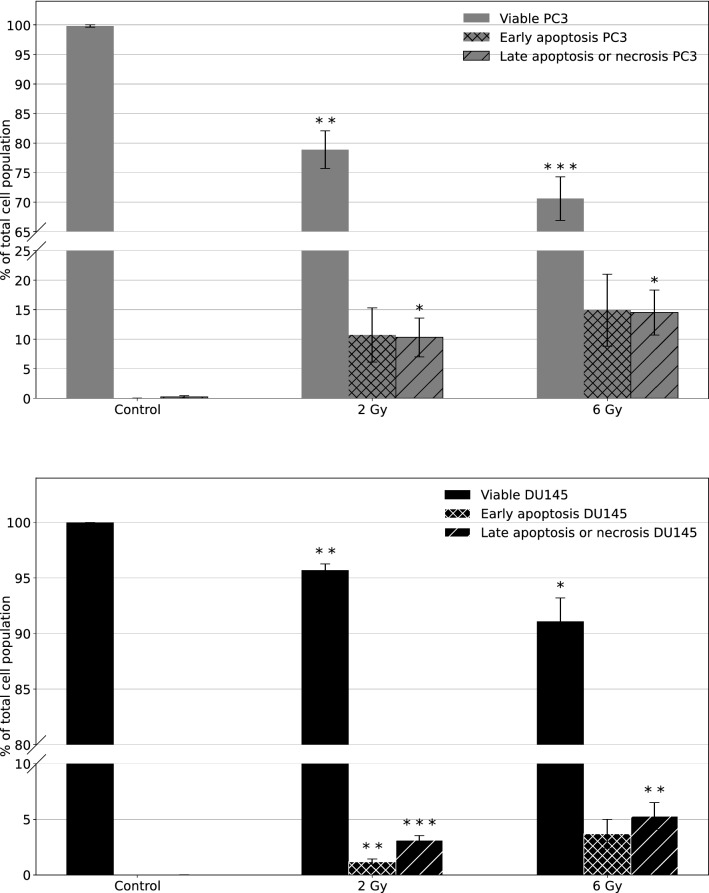
The dose-effect of alpha radiation on viable cells and cells undergoing early and late apoptosis or necrosis in PC3 (upper panel) and DU145 (lower panel) cells as detected by flow cytometry 96 h after irradiation and averaged from 3 independent experiments undergoing for 0 Gy (control), 2 Gy, and 6 Gy radiation wherein each radiation dose had two replicates. Data are presented as $\%$ of viable cells, cells at an early stage of apoptosis, and as $\%$ of cells at late-stage apoptosis or necrotic cells. Error bars represent  ± 1 standard deviation, $^{\star \star \star }$ *p* $\le$ 0.001, $^{\star \star }$ *p* $\le$ 0.01 and $^{\star }$ *p* $\le$ 0.05 is calculated as compared to control, non-irradiated cells and marked as $^{\star }$. Staining FITC-Annexin V/7-ADD flow cytometry: viable cells (Annexin V-FITC negative and 7-ADD negative staining), early apoptotic cells (Annexin V-FITC positive and 7-ADD negative staining), late-stage apoptotic cells or necrotic cells (Annexin V-FITC positive and 7-ADD positive and Annexin V-FITC negative and 7-ADD positive staining, respectively)

Annexin V-FITC/7-AAD flow cytometry was used to study the fundamental mechanism of cell death induction and the effects of alpha particles on viable cells and early and late stage apoptosis. The number of viable cells and those undergoing early and late stage apoptosis or necrosis was averaged for all experiments (Fig. 2) which were based on diagrams of FITC-Annexin V/7-ADD flow cytometry (supplementary materials).

Annexin V-FITC/7-AAD staining of tumor cells showed an increased amount of early apoptotic and late apoptotic or necrotic cells as a function of radiation dose for both PC3 and DU145 cells. The difference in the number of early apoptotic cells was higher for PC3 (10.7 ± 4.6 and 14.9 ± 6.1) than for DU145 (1.19 ± 0.24 and 3.7 ± 1.3) cells for 2 Gy and 6 Gy, respectively. The same observation was noted for late apoptotic or necrotic cells (when irradiated with 2 Gy: 10.3 ± 3.3 and 3.12 ± 0.42, and when irradiated with 6 Gy: 14.5 ± 3.8 and 5.3 ± 1.2 for PC3 and DU145 cells, respectively). The level of statistical significance for the number of dead cells for control and 2 Gy irradiated samples was greater for DU145 (*p* $\le$ 0.001 and *p* $\le$ 0.01, for late and early apoptosis, respectively) than for PC3 cells, where a statistically significant difference was observed only for late apoptosis (*p* $\le$ 0.05) compared to control cells. Radiation-induced accumulation of apoptotic cells was most prominent after exposure to 6 Gy (*p* $\le$ 0.01 for DU145 cells and *p* $\le$ 0.05 for PC3 cells for late-stage apoptosis). Additionally, PC3 cells are more radiosensitive than DU145 cells, which is supported by the increased number of dead cells (around 3 times more) after alpha irradiation for PC3.

### LDH

**Fig. 3:**
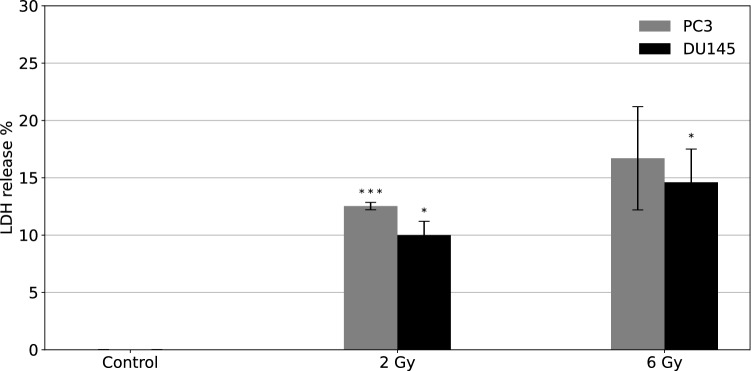
Determination of cell death measured 96 h after irradiation using LDH assay for PC3 (marked in gray) and DU145 (marked in black) as a function of dose delivered by alpha particles. The mean values are calculated based on 3 independent experiments with two replicates for each radiation dose. The error bars represent  ± 1 standard deviation, $^{\star \star \star }$ *p* $\le$ 0.001 and $^{\star }$ *p* $\le$ 0.05 as compared to control cells

The results of the LDH assay performed for PC3 and DU145 are shown in (Fig. 3).

The LDH release was used as a radioresistance marker for the PC3 and DU145 cells. LDH results demonstrated that cell membrane damage increased as a function of IR dose. The difference in the level of LDH was higher for PC3 (12.53 ± 0.32 and 16.7 ± 4.5) than for DU145 (10.0 ± 1.2 and 14.6 ± 2.9) cells for 2 and 6 Gy, respectively. While a statistically significant difference in LDH release for DU145 cells was observed for both doses (*p* $\le$ 0.05 for 2 Gy and 6 Gy), for PC3 cells statistical significance was only observed for 2 Gy (*p* $\le$ 0.001) treatment. No statistical difference was seen for 6 Gy treated PC3 cells because of greater variance.

### IL-6

**Fig. 4:**
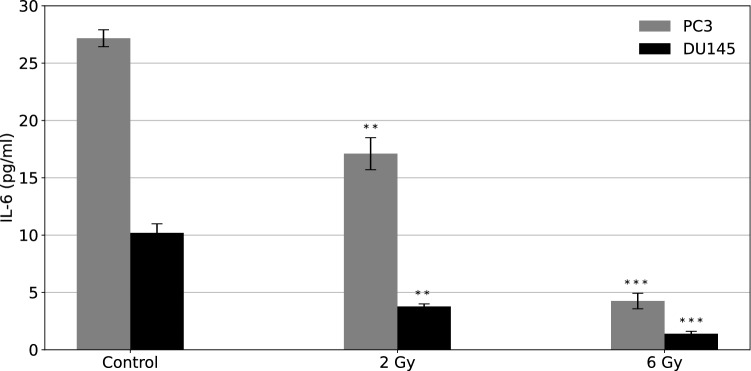
IL-6 concentration measured 96 h after irradiation for PC3 (marked in grey) and DU145 (marked in black) cells as a function of dose delivered by alpha radiation. The mean values are calculated based on 3 independent experiments with two replicates for each radiation dose. The error bars represent  ±  1 standard deviation, $^{\star \star \star }$ *p* $\le$ 0.001 and $^{\star \star }$ *p* $\le$ 0.01 as compared to control

The concentration of IL-6 in the culture supernatant was measured for PC3 and DU145 cells exposed to alpha radiation and shown in (Fig. 4).

The concentration of IL-6, which is indicative of the presence of inflammation, decreased with increased radiation dose for both PC3 and DU145 cells. The observed differences between irradiated and control cells were statistically significant (*p* $\le$ 0.01 for 2 Gy and *p* $\le$ 0.001 for 6 Gy). The IL-6 concentration was higher for PC3 cells than in DU145 cells by a factor of 3 (for 0 and 6 Gy irradiation) and even higher for 2 Gy treatment (around 4.5 times more).

### NTA

**Fig. 5:**
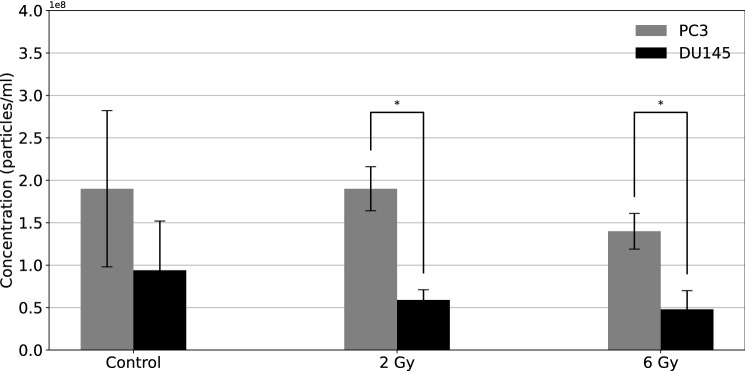
The concentration of exosomes isolated 96 h after irradiation from PC3 (marked in grey) and DU145 (marked in black) cells as a function of alpha radiation dose. The mean values are calculated based on 3 independent experiments with two replicates for each radiation dose. The error bars represent  ± 1 standard deviation, $^{\star }$ *p* $\le$ 0.05 as compared between PC3 and DU145 cells for a given radiation dose

The exosomes isolated from PC3 and DU145 cells exposed to 0 Gy, 2 Gy, and 6 Gy of alpha radiation were counted with NTA and shown in (Fig. 5). Concentration was calculated as the number of detected vesicles per 1 mL of supernatant.

**Fig. 6:**
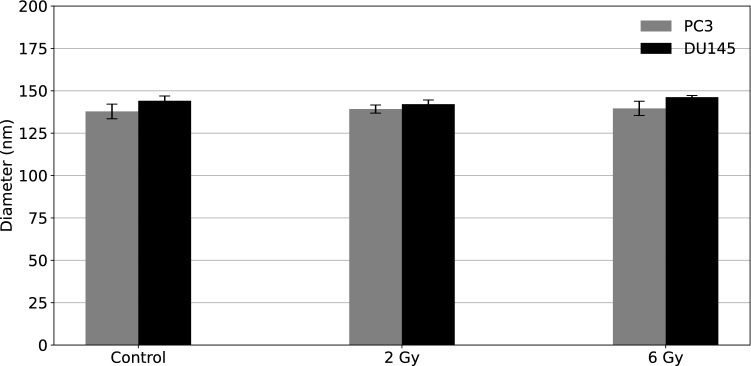
Exosome size characteristics for PC3 (marked in grey) and DU145 (marked in black) cells as a function of alpha particle dose. The mean values and their standard deviations were calculated for exosome hydrodynamic diameter based on 3 independent experiments with two replicates for each radiation dose

A gradual decrease in exosome concentration was observed in 2 Gy and 6 Gy alpha-particle-irradiated samples compared to the control samples. The number of exosomes isolated from PC3 cells was greater than those isolated from DU145 cells (2 times more for control samples, and about 3 times more for exosomes isolated from the irradiated cells). The number of exosomes secreted from PC3 and DU145 cell lines was statistically different for both 2 Gy (*p* = 0.011) and 6 Gy (*p* = 0.042) radiation treatments. In contrast, control, non-irradiated cells from the two prostate cancer cell lines exhibited no statistically significant difference in the number of exosomes. Additionally, the exosome sizes were measured and presented in (Fig. 6).

According to the NTA measurements of the exosomes extracted from the human prostate cancer cell lines, the isolated vesicles were 137–146 nm in diameter, which corresponds to the size of exosomes. No correlation was observed between the nanoparticle size and the dose delivered to the cells. Moreover, no significant differences were found between the average diameters of the exosomes and the radiation dose (*p* > 0.05).

## Discussion

The aim of this work was to investigate the effect of high LET radiation on human prostate cancer cells and exosome secretion from these cells. In the present study, we selected the doses of 2 and 6 Gy as representative of the 2 Gy daily clinical fractions given during RT and the 3–10 Gy doses given in palliative RT (RCR 2019). Two human prostate cancer cell lines, PC3 and DU145, were exposed to alpha radiation. PC3 cells are known to be more tumorigenic and have a higher metastatic potential (Webber et al. 1997; Katsogiannou et al. 2019) compared to DU145 cells.

Apoptotic cell death plays a prominent role in the death of normal and malignant prostate tissue. One of the ways in which cancer cells appear resistant to RT or chemotherapy is disruption of the pathways that lead to apoptosis (Vucic et al. 2006). Furthermore, when comparing the number of dead cells in the total cell population, DU145 cells exhibit a greater magnitude of radioresistance than PC3 cells. This finding was consistent with a previous study (Jayakumar et al. 2014) where human prostate cancer cell lines were irradiated using $^{60}$Co gamma rays.

LDH release was increased in irradiated cells compared to control cells for both cell lines. The difference in LDH activity between PC3 and DU145 cells was observed previously in non-irradiated cells (Cavallari et al. 2020) and PC3 cells exhibited greater LDH concentrations compared to DU145 cells. PC3 cells are also much more glycolytic than DU145 cells. Our results demonstrated that there was a slightly greater release of LDH in PC3 than DU145 cells when cells were exposed to alpha radiation. In the context of photon RT treatment, high LDH expression is considered a poor prognostic factor associated with radioresistance, and its impact on survival fraction was studied for many cancer cell types (Liu et al. 2021). Low LET radiation-induced accumulation of LDH activity was studied in different cell lines [e.g. in Jurkat cells using Cs-137 (Ferranti et al. 2020) and in thymus extracts of mice using X-rays (Hori et al. 1968)]. However, the influence of IR (neither low nor high LET) on the LDH release in human prostate cancer cell lines was not explored. Thus, using the LDH assay for PC3 and DU145 cells irradiated with alpha particles emitted from Am-241 we obtained the first results on LDH activity as a function of radiation dose for this type of cancer cell. No noticeable differences were found between the PC3 and DU145 cell lines based on the apoptosis study and the LDH assay. However, the annexin/PI staining in the flow cytometry experiment suggested that PC3 cells were more radiosensitive. Densely ionizing alpha radiation interacting with the cell causes double-stranded breaks in DNA. We observed that alpha radiation did not severely damage the cell membrane, but irradiation did activate the process of apoptosis in cells. The observed increase in the LDH activity might be influenced by the presence of necrotic processes within the tumor tissue as suggested by (Forkasiewicz et al. 2020).

The concentration of IL-6 in the culture supernatant measured for PC3 and DU145 cells exposed to alpha radiation decreased as a function of dose. The level of IL-6 secretions decreased following high LET radiation treatment. However, this trend was not observed in cells exposed to low LET radiation. IL-6 expression measured using Western blotting and ELISA by (Wu et al. 2013) was enhanced in HR prostate cancer cells after X-ray irradiation. Moreover, increased concentrations of IL-6 suppressed low LET radiation-induced cell death in oral squamous cell carcinoma (Matsuoka et al. 2016), which showed the biological roles of IL-6 on radiosensitivity.

In sum, the number of apoptotic cells increased with the amount of alpha radiation and a greater release of LDH corresponded to an increased level of cell membrane damage. On the other hand, a decrease in IL-6 secretion could be related to the decreased number of living cells not killed by alpha radiation.

The influence of IR on exosome release was observed for the first time (Yu et al. 2006) when the human epithelial lung cancer cell line H460 was exposed to gamma irradiation. Cell cultures were irradiated with 5 Gy (CIS BioInternational IBL 437C $^{137}$Cs, dose rate 0.49 Gy/min). In this study, exosome secretion by cells was regulated by the p53 protein, which responds to stress signals. Moreover, TSAP6, a p53-regulated gene product, enhances exosome production in cells undergoing a p53 response to stress. In irradiated, p53-competent cells, increased levels of exosome release were confirmed in aneuploid immortal HaCaT keratinocytes (Jella et al. 2014), MCF7 human breast adenocarcinoma cells (Al-Mayah et al. 2015), and LNCaP, 22Rv1, and DU145 human prostate cancer cell lines (Lehmann et al. 2008). HaCaT cells were irradiated at 0.005, 0.05, and 0.5 Gy using gamma irradiation produced from a $^{60}$Co teletherapy unit. Exosomes were measured using light scattering analysis (LSA) and the LSA exhibited an increased concentration of exosomes as a function of radiation dose (Jella et al. 2014). In studies on human breast adenocarcinoma (Al-Mayah et al. 2015), in which MCF-7 cells were exposed to 2 Gy X-rays, the obtained results showed that exosomes played an important role in mediating non-targeted effects (NTE) of IR. A similar phenomenon was observed in studies of exosomes derived from LNCaP, 22Rv1, and DU145 cells (Lehmann et al. 2008) after gamma irradiation. Their results suggested that exosomes could transfer cargos between cells through a novel mechanism, which could be recruited to increase exosome release during accelerated and replicative cellular senescence. In 22Rv1 cells, irradiation-induced senescence was related to an increased release of B7-H3-positive exosomes that were free of contamination by cytosolic or mitochondrial proteins. The difference in exosome concentration between non-irradiated samples and irradiated samples was observed in (Arscott et al. 2013). Furthermore, they showed higher concentrations of exosomes released by glioblastoma cells which were treated with an X-ray source. Moreover, the measurements of the exosomal cargo by (Jelonek et al. 2015) showed an increase of proteins in exosomes from irradiated cells with respect to control samples. In this work, exosomes were isolated from human squamous head and neck cells (FaDu), which were irradiated with 2 Gy using the linear accelerator (Clinac 600, 6 MeV photons). The exosome studies performed for low LET irradiation of U87 glioma cells (Mrowczynski et al. 2018) and LN18, U251, and U87MG cells (Arscott et al. 2013) demonstrated the increase in exosome abundance compared to the release of exosomes from control cells. In the present work, we evaluated whether the stress of IR alters the concentration and size of exosomes released from human prostate cancer cells. Our results obtained with NTA revealed that due to large variances there was no statistically significant difference in the number of exosomes from PC3 and DU145 with increasing doses of alpha radiation. However, the concentration of exosomes isolated from PC3 cells was higher than the more radioresistant DU145 cell line. We also observed a decrease in the exosome secretion as a function of the radiation dose delivered to the cells (either PC3 or DU145) by alpha particles, which was consistent with a study (Elbakrawy et al. 2021) where human fetal lung fibroblast HF19 cells were irradiated using $^{238}$Pu alpha-particle source.

The average diameter of exosomes isolated from PC3 and DU145, which were exposed to alpha radiation, did not depend on the radiation dose delivered to the cells, and no significant differences were found between control and irradiated cells, which corroborated previous work (Elbakrawy et al. 2021). Elbakrawy et al. studied characteristics of exosomes secreted from HF19 after alpha irradiation. However, exosomes isolated from cells treated with low LET radiation did exhibit some difference between irradiated and non-irradiated, control cells. For example, in studies on human breast cancer cells (Jabbari et al. 2019) the size of exosomes secreted by irradiated cells increased with an increasing dose of X-rays. On the other hand, in studies of exosomes derived from endothelial cells (Bagheri et al. 2018) no size-dose dependency was observed after cell exposure to the low-level laser.

Although the direct experiments using photons were not conducted by our group, the differences between low and high LET radiation can be observed indirectly for apoptosis, LDH, IL-6, and exosome concentration studies. IR reduces the number of viable cells of PC3 and DU145 when exposed to gamma radiation (Jayakumar et al. 2014). Increased levels of IL-6 suppressed low LET radiation-induced cell death in oral squamous cell carcinoma (Matsuoka et al. 2016). An increase in exosome concentration was observed in U87MG cells, which have similar size, doubling time, and enzymatic properties as DU145 (Arscott et al. 2013). In the case of studies using low LET radiation, the works cited by us show that the concentration of exosomes increases with increasing dose. We observed an opposite effect for studies using alpha particles (high LET), but it is consistent with previous work (Elbakrawy et al. 2021). Individual cells exhibit different levels of radiosensitivity, which affects the concentration of exosomes secreted from cells exposed to IR. While the exosome concentration decreased with the dose delivered by alpha particles, it increased with the increasing dose of X-rays. However, changes in exosome concentration are different for different types of radiation. The change in concentration measured with respect to the control sample is the quantity which matters. Therefore, exosomes could be an efficient biomarker for alpha and gamma radiation exposure.

## Conclusion

The role of IR in exosome secretion remains largely unclear. Since the biological effectiveness of IR depends on the radiation quality used for cell exposure, the exosome-mediated signaling may also differ for sparse (low LET) and dense (high LET) IR. We have evaluated the cellular response of human prostate cancer cells exposed to alpha radiation and the corresponding effects on the concentration and size of isolated exosomes. This study shows that alpha radiation affects PC3 and DU145 similarly when taking into account apoptotic and necrotic cells and measuring the concentration of LDH and IL-6. There is no difference in exosome size isolated from PC3 and DU145 cells with increasing radiation doses delivered by alpha particles. As a general rule, cancer cell lines secrete more exosomes than normal cells. In our work, a decrease in exosome concentration was observed (without statistical significance) as a function of the radiation dose absorbed in both human prostate cancer cell lines we studied. Furthermore, we found that the more radioresistant cell line (DU145) secreted fewer exosomes than PC3 cells (the radiosensitive cell line). Exosomes contain various cargoes and are involved in cell-to-cell communication independently of IR. Furthermore, exosomes are generally considered to be efficient biomarkers for new treatments or/and drug development. For this reason, in our future work, we will determine whether alpha particles induce changes in the profile of exosomes released from PC3 and DU145 cells after IR exposure to variable doses. We predict that more radioresistant cells may produce exosomes carrying a different cargo. For the first part of this study, we utilized alpha radiation because alpha particles ionize densely, similar to protons, whose clinical use is increasingly common alongside conventional therapies. We plan to conduct further experiments using the same parameters as the current study but utilizing X-ray radiation instead of alpha particles. In this manner, we can draw meaningful conclusions about the impact of different radiation sources on exosomes secreted by PC3 and DU145 cells and determine whether exosomes are equally efficient biomarkers of high and/or low LET radiation in the case of human prostate cancer cell lines with different radiosensitivities.

## Supplementary Information

Below is the link to the electronic supplementary material.

Supplementary file1 (PDF 685 kb)

## Data Availability

Data will be available if requested.
