# Supplementary material for: Exosome secretion and cellular response of DU145 and PC3 after exposure to alpha radiation
Source: Radiat Environ Biophys. 2022 Sep 13;61(4):639–50. doi: 10.1007/s00411-022-00991-5 (PMC9630248; doi:10.1007/s00411-022-00991-5)
Supplement: Supplementary file 1 — Supplementary file1 (PDF 685 kb) [file 411_2022_991_MOESM1_ESM.pdf]

Appendix: Supplementary Material

A. Exosome confirmation using NTA with antibodies

The exosome confirmation was performed using the NTA measurement with antibodies -CD9, -CD81, and -CD63 (Gardiner et al, 2013 and Ludwig et al, 2019a) and it is shown in Fig. A. The tetraspanins of individual PC3 and DU145 exosomes were analyzed by both NTA and total internal reflection fluorescence microscopy. Fluorescence-labeled membrane proteins on exosomes were detected by comparing scattering and fluorescent images.

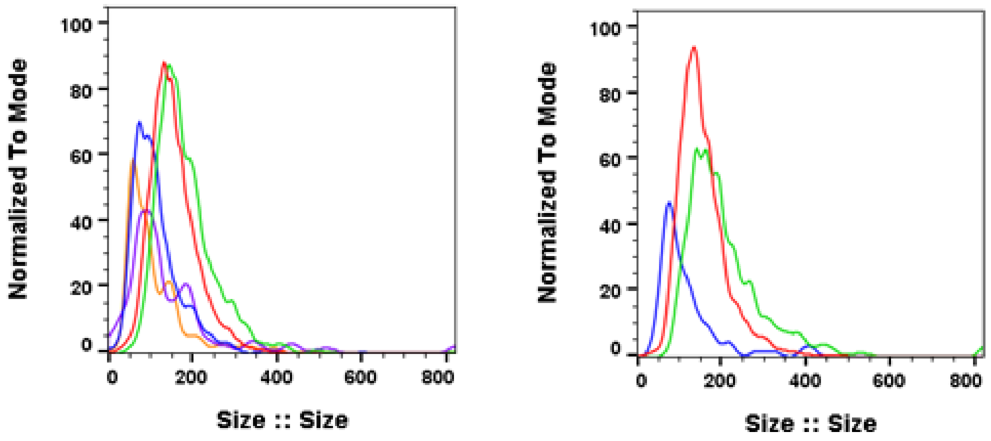

Figure A: The identity of exosomes using antibodies: anti-CD9 (blue), -CD63 (orange), and -CD81 (violet) by a fluorescence-based NTA system and by light scattering (red) for the characterization of both the size and membrane protein expression. The measurements were performed for PC3 cells (right panel) and DU145 (left panel).

A cell mask (CMDR), specifically lipophilic membrane dyes such as Cell Mask© Deep Red (CMDR, Thermo Fisher, USA) is shown by the green line.

Exosomes are defined as a subset of extracellular vesicles (EVs) sized at 30 to 150 nm. The method in our study utilizes size exclusion chromatography (SEC) for recovery of exosomes from cell-line supernatants. Tetraspanins: CD9, CD63, CD81 (which we confirmed by NTA) are considered "exosome markers".

The camera control interface is responsible for setting the camera controls manually (basic and advanced).

Basic mode – camera level Determines the brightness of the image (using a combination of shutter and gain used in advanced mode)

Advanced mode - Shutter Determines the length of time the camera electronic shutter is open and therefore how much light is captured from the particles.

Advanced mode - Gain This setting increases the sensitivity of the camera and should be increased to allow the smallest particles present in the sample to be made visible and therefore capable of being tracked and analysed (Bachurski et al, 2019).

Vesicles positive for Surface markers seem to be a smaller supopulation of all vesicles (scatter mode). The exosomal marker (CD9) is most common in fraction #4, which indicates a high exosome content in this fraction (Ludwig et al, 2019b).

References

1. Bachurski, D., et al., Extracellular vesicle measurements with nanoparticle tracking analysis - An accuracy and repeatability comparison between NanoSight NS300 and ZetaView. J Extracell Vesicles, 2019. **8**(1): p. 1596016.
2. Ludwig, N., et al., Optimization of cell culture conditions for exosome isolation using mini-size exclusion chromatography (mini-SEC). Exp Cell Res, 2019b. **378**(2): p. 149-157.

B. Diagrams of FITC-Annexin V/7-ADD flow cytometry

To interpret the fundamental mechanism of cell death induction, the effect of alpha particles on early and late apoptosis using Annexin V-FITC/7-AAD flow cytometry analysis was studied. The early and late apoptosis or necrosis averaged for all experiments were shown for PC3 in Fig. 1B based on diagrams of FITC-Annexin V/7-ADD flow cytometry. The same data measured for the DU145 cell line is presented in Fig. 2B.

A

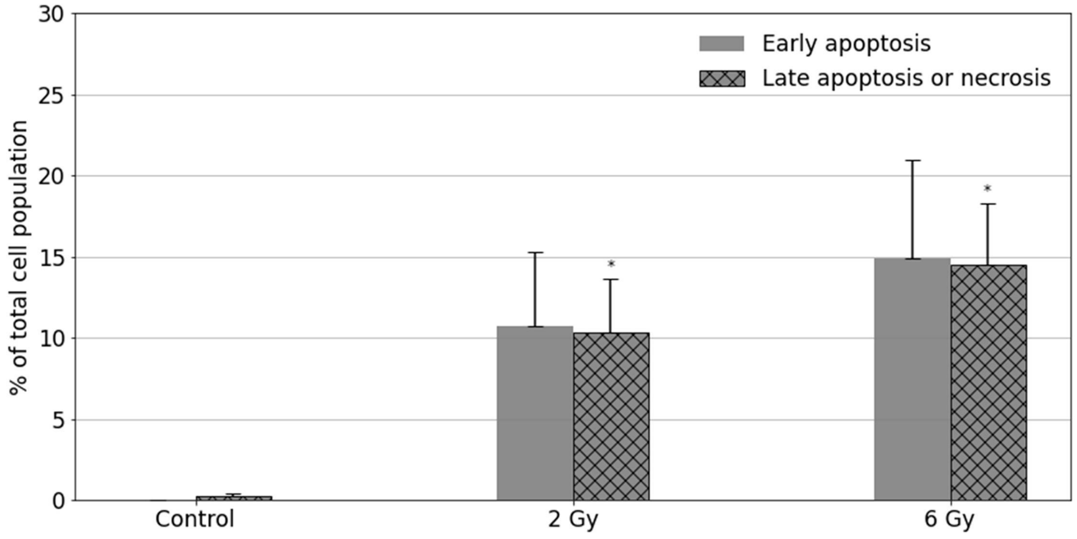

B

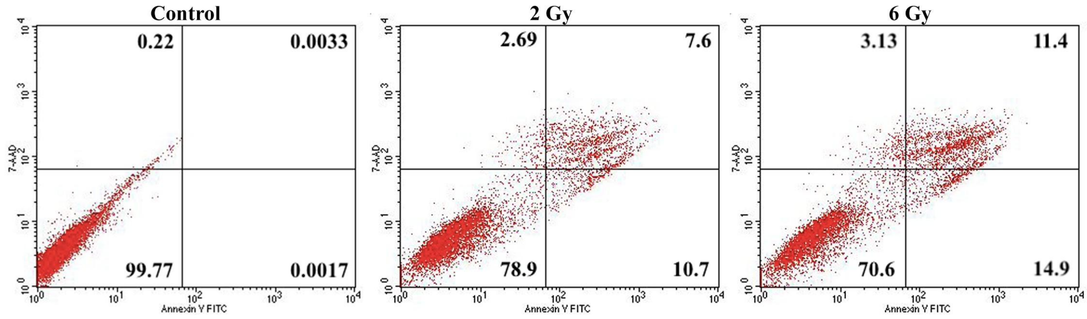

Figure 1B. The dose-effect of alpha radiation on early and late apoptosis or necrosis in PC3 cells as detected 96 hours after irradiation by flow cytometry and averaged from 3 independent experiments, each in two replicants performed for 0 Gy (control), 2 Gy, and 6 Gy.

(A) Data are presented as % of cells at an early stage of apoptosis and as % of cells at late-stage apoptosis or necrotic cells. Error bars represent standard deviation, $p \leq 0.05$ is calculated as compared to control and marked as *.

(B) Diagrams of FITC-Annexin V/7-ADD flow cytometry. The lower left quadrant represents viable cells (Annexin V-FITC negative and 7-ADD negative staining). The lower right quadrant represents early apoptotic cells (Annexin V-FITC positive and 7-ADD negative staining). The upper right and upper left quadrants contain late-stage apoptotic cells or necrotic cells (Annexin V-FITC positive and 7-ADD positive and Annexin V-FITC negative and 7-ADD positive staining, respectively).

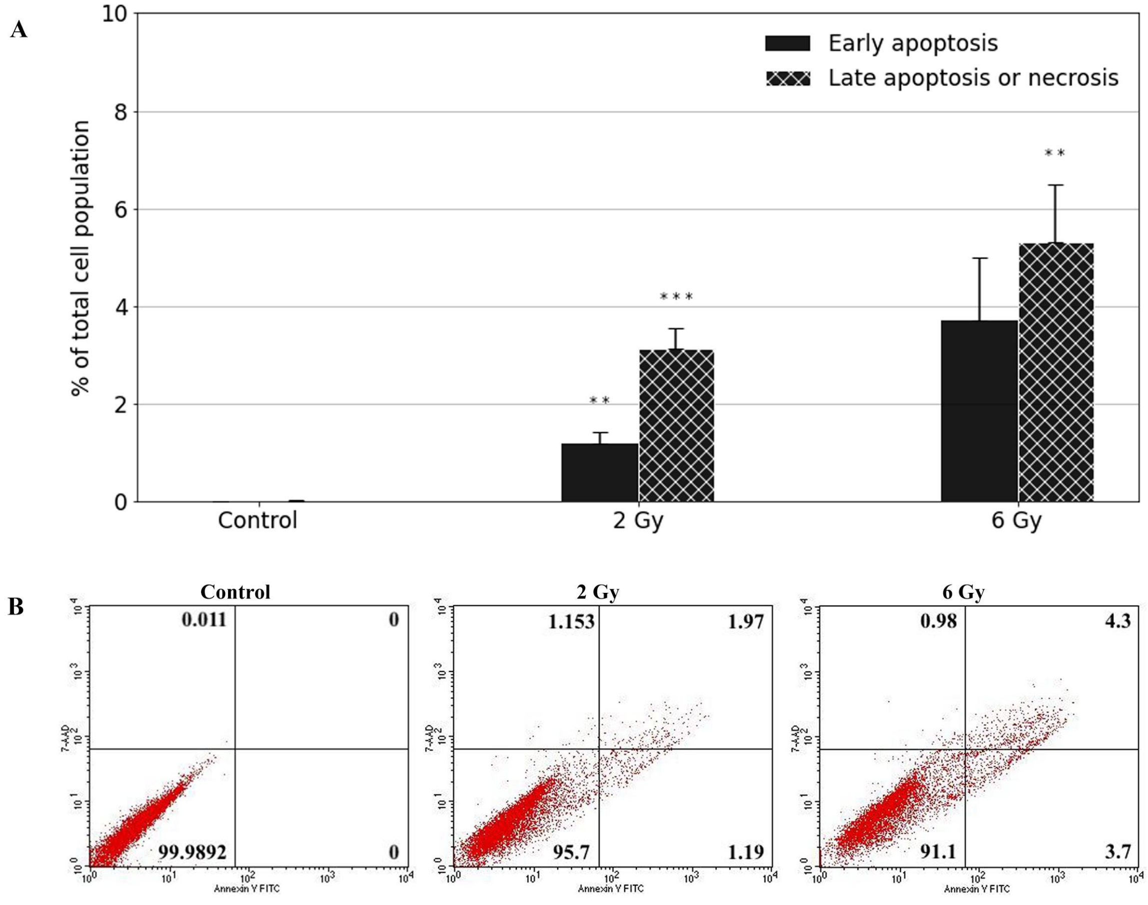

Figure 2B. The dose-effect of alpha radiation on early and late apoptosis or necrosis in DU145 cells as detected 96 hours after irradiation by flow cytometry averaged from 3 independent experiments, each in two replicants performed for 0 Gy (control), 2 Gy, and 6 Gy.

(A) Data are presented as % of cells at an early stage of apoptosis and as % of cells at late-stage apoptosis or necrotic cells. Error bars represent standard deviation, $p \leq 0.001$ is marked as ***, $p \leq 0.01$ is marked as **, *p*-values are calculated as compared to control.

(B) Diagrams of FITC-Annexin V/7-ADD flow cytometry. The lower left quadrant represents viable cells (Annexin V-FITC negative and 7-ADD negative staining). The lower right quadrant represents

early apoptotic cells (Annexin V-FITC positive and 7-ADD negative staining). The upper right and upper left quadrants contain late-stage apoptotic cells or necrotic cells (Annexin V-FITC positive and 7-ADD positive and Annexin V-FITC negative and 7-ADD positive staining, respectively).
